# Childhood neglect predicts the course of major depression in a tertiary care sample: a follow-up study

**DOI:** 10.1186/s12888-017-1270-x

**Published:** 2017-03-28

**Authors:** Sabrina Paterniti, Irit Sterner, Christine Caldwell, Jean-Claude Bisserbe

**Affiliations:** 1 Royal Ottawa Mental Health Centre, 1145 Carling Avenue, Ottawa, ON K1Z 7K4 Canada; 2 Department of Psychiatry, University of Ottawa, Ottawa, ON Canada; 3 Department of Psychology, University of Ottawa, Ottawa, ON Canada; 4 Université Paris Est Créteil, Paris, France

**Keywords:** Depression, Risk factors, Childhood maltreatment, Longitudinal study

## Abstract

**Background:**

The course of depression is poorer in clinical settings than in the general population. Several predictors have been studied and there is growing evidence that a history of childhood maltreatment consistently predicts a poorer course of depression.

**Methods:**

Between 2008 and 2012, we assessed 238 individuals suffering from a current episode of major depression. Fifty percent of these (*N* = 119) participated in a follow-up study conducted between 2012 and 2014 that assessed sociodemographic and clinical variables, the history of childhood abuse and neglect (using the Adverse Childhood Experience questionnaire), and the course of depression between baseline and follow-up interview (using the Life Chart method). The Structured Clinical Interview for DSM-IV-TR was used to assess diagnosis at baseline and follow-up interview. Statistical analyses used the life table survival method and Cox proportional hazard regression tests.

**Results:**

Among 119 participants, 45.4% did not recover or remit during the follow-up period. The median time to remission or recovery was 28.9 months and the median time to the first recurrence was 25.7 months. Not being married, a chronic index depressive episode, comorbidity with an anxiety disorder, and a childhood history of physical neglect independently predicted a slower time to remission or recovery. The presence of three or more previous depression episodes and a childhood history of emotional neglect were independent predictors of depressive recurrences.

**Conclusions:**

Childhood emotional and physical neglect predict a less favorable course of depression. The effect of childhood neglect on the course of depression was independent of sociodemographic and clinical variables.

## Background

The course of depression is poorer in psychiatric settings than in the general population or primary care (see reviews [1–3]). Long-term follow-up studies (>10 years) examining the course of major depressive disorder (MDD) have identified rates of stable recovery without recurrences in 47% of patients with a first episode of depression in the general population [4], in 35% of patients treated in primary care [5], and in 20%–30% of patients in specialized care [6, 7]. These differences in prognosis can be explained by the higher severity of depression in tertiary care patients. Tertiary care patients are referred to specialized services because the severity of their symptomatology can require hospitalization, because of the chronic or highly recurrent course of their mood episodes, or because they present with treatment-resistant depressive symptoms [2]. We know that the above clinical characteristics predict poor prognosis of depression: in cohort studies, longer index episode length, higher number of previous episodes, and higher index episode severity predict poorer prognosis in terms of chronic or recurrent course of depression (for reviews of the literature see [8, 9]). Data from the Sequenced Treatment Alternatives to Relieve Depression (STAR*D) study indicate that patients with symptoms resistant to antidepressant trials had lower remission rates and higher relapse rates during the follow-up. The greater the number of trials failed, the greater the risk of poor prognosis [10].

Studying the course of depression in a tertiary care population has both advantages and disadvantages. A tertiary care population, including patients with treatment-resistant depression, is most likely to suffer from severe functional impairment and negative impact on quality of life, resulting in high costs from utilization of mental health services. Targeting this population through effective treatment could contribute to reducing the global burden of depression [11, 12]. However, as depressed tertiary care patients are not representative of depressed patients in general, results cannot be generalized.

Some studies have examined the course of depression in treatment-resistant patients, who show a poorer course of depression than individuals in the general population [6, 13–15]. However, the cohorts that these studies examined were relatively old, as they were in their 70s [6, 14], 80s [13], or early 90s [15]. Given the recent development of pharmacological tools, particularly the use of atypical antipsychotics for treatment-resistant depression [16], it is important to examine the course of depression in a more recently recruited tertiary care sample.

The outcome of depression can be determined in several different ways that combine the domains of symptoms, functional state (such as psychosocial functioning and quality of life), and pathophysiological changes [17]. Some authors focus only on depression symptoms to categorize the outcome as “remission, recovery and recurrence” [18]. Although functional status is an important part of the outcome, its inclusion in the definition of outcome may be problematic, particularly in tertiary care patients. Many treatment-resistant depressed patients present with associated medical conditions, which may affect functional status independently of depression. Furthermore, recovery and remission are usually associated with a return to premorbid levels of functioning. For these reasons, the American College of Neuropsychopharmacology Task Force on Response and Remission in Major Depressive Disorders recommend not including the assessment of functional status in the definition of outcome [19].

There is increasing evidence that childhood abuse and neglect affect the course of adult psychiatric disorders, particularly anxiety and depressive disorders (for reviews of the literature see [20, 21]). Interestingly, Brown et al*.* [22] found that samples of depressed subjects drawn from clinical populations (inpatients, day patients, or outpatients treated in hospital psychiatric departments) had a higher prevalence of childhood sexual or physical abuse or parental indifference than samples drawn from the general population.

Several studies have examined the effect of childhood adverse experiences on the course of MDD and most have found a substantial association between a history of childhood maltreatment and the course of depression in adulthood. According to the American Center for Disease Control [23], childhood maltreatment is defined as abuse or neglect of an individual under 18 years by any person in a custodian role. One of the forms of neglect included in this definition is exposure to violent environments. In a recent meta-analysis, Nanni et al*.* [20] found similar effect sizes for the effect of childhood maltreatment on the risk of depressive recurrences (odds ratio [OR] = 2.2, 95% confidence intervals [CI] = 1.6–3.1) and persistence of depressive symptoms (OR =2.3, 95% CI = 1.6–3.3). Nanni et al*.* also found that the ORs of most of the studies included in the meta-analysis indicated a two- or threefold higher risk of persistent or recurrent depression when there was a history of childhood maltreatment, although the studies showed quite a large range of minimum and maximum ORs: between 1.3 [24] and 14.9 [25].

Most previous studies have assessed the presence of childhood maltreatment retrospectively in adulthood; one problem with this is the possibility of recall bias, as depressed adults may recall their childhood in a more negative way. However, at least two prospective studies indicate that childhood maltreatment, assessed in childhood or adolescence, predicts the course of depression in adulthood [26, 27].

Sociodemographic variables, family context, and psychological and clinical factors have been considered as possible confounders of the relationship between childhood maltreatment and course of depression, especially in the most recent studies. The association between childhood maltreatment and course of depression seems independent of age, race, gender, education, and marital status, which have been used as adjustment factors in several studies [28–30]. Interpersonal difficulties in adulthood only partially explain the association between childhood maltreatment and chronicity of depression, suggesting that childhood maltreatment directly affects the course of depression. This association is not completely explained by the possible impact of a history of childhood maltreatment on interpersonal difficulties, which are also a predictor of chronic depression [31]. Similarly, Ritchie et al*.* [32] found that the association of traumatic events in childhood with the persistence of depression was independent of recent life events. Interestingly, two studies have shown that parental mental health issues only partially explain the association between childhood maltreatment and course of depression [26, 33]. It is therefore unlikely that the observed association is caused only by a familial predisposition to depression [34]. This also indicates that the common shared familial genetic predisposition to depression cannot completely explain the relationship between childhood maltreatment and course of depression. Moreover, a history of family adversity in childhood (parental discord, separation from parents) does not predict the course of depression, whereas a history of childhood abuse does [26]. Finally, some research indicates that the association between childhood maltreatment and course of depression is independent of clinical variables, such as age at onset of depression [28, 30], anxious personality or conduct problems in childhood, personality traits in adolescence [35], and comorbid anxiety [30].

In summary, there are fairly consistent findings linking childhood maltreatment to the course of depression, with few exceptions [24, 36]. Furthermore, the association between childhood maltreatment and course of depression seems independent of a large range of factors.

To our knowledge, few studies have considered the role of childhood maltreatment in predicting the course of depression in tertiary care samples, and these have only considered a limited number of possible confounders [22, 36, 37].

As the course of depression is worse in psychiatric settings than in the general population, it is important to replicate these findings in clinical samples, taking into account the possible confounding effects of various demographic and clinical variables known to affect the course of depression.

The goals of the present study are 1) to investigate the course of depression in a sample of tertiary care depressed patients using a 2–5-year naturalistic follow-up and 2) to examine the independent role of clinical variables, sociodemographic variables, and childhood abuse and neglect in predicting the course of severe treatment-resistant depression.

Our hypotheses are as follows: 1) the course of depression in a treatment-resistant sample is poorer than in the general population, with slower remission time and higher recurrence rate and 2) the presence of childhood abuse and neglect predicts a poorer course of depression in a tertiary care sample.

## Methods

This study was conducted in the context of the Assessment and Treatment Clinic (ATC), an outpatient service established between December 2006 and December 2012 within the framework of the Royal Ottawa Mental Health Centre’s (ROMHC) Mood Disorders Program.

The ROMHC Mood Disorders Program provides specialized tertiary care services to the adult population of the Local Health Integration Network district, Ottawa, Ontario, Canada (estimated population aged 18–65 in 2009: 600,000). The program targets high-risk mood disorder patients; namely, “individuals with serious, complex, and/or rare mental disorders, who present multiple and complex needs, and whose treatment requirements cannot be met in the first line or at the more intensive levels of service” (Ontario Ministry of Health, May 2005. Making It Happen: Operational Framework for the Delivery of Mental Health Services and Support). The ATC includes patients suffering from primary mood disorders whose treatment resistance or severity requires the intervention of the outpatient multidisciplinary team. As such, these patients need more intensive and prolonged care resources. Patients with an active substance use disorder are usually not included but are referred to a specialized program. The selection of cases for ATC assessment has been described in a previous paper [38].

### Baseline evaluation

Following the initial assessment by a psychiatrist, the subjects underwent a clinical interview with a nurse, a social worker, an occupational therapist, and a psychologist. The psychologist administered the Structured Clinical Interview for DSM-IV-TR (SCID) [39] to assess Axis I diagnoses. The SCID Mood Disorder and Post Traumatic Stress Disorder modules were completed for every patient*.* The history of mood disorder was also examined using the SCID, obtaining information related to the age of onset of MDD, the length and severity of the index major depressive episode (MDE), and the number of previous MDEs. The SCID screen was administered to all patients. When the SCID screening indicated an anxiety, psychotic, or eating disorder, these modules were administered as well. Substance abuse and dependence were flagged, based on the chart review, SCID screening, and the patient’s report during the interdisciplinary assessment. The nurse collected information related to the medical conditions based on the participant’s report, the referral form information, and (when available) the clinical chart. The nurse collected psychiatric family history using the question “Does anyone in your family have a history of mental illness, alcohol abuse, or drug abuse?” Additional details about the kind of illness or substance abuse exhibited by the family member, such as if the condition had been diagnosed and/or treated, were also collected. The social worker obtained details of the family environment, including the presence of mental illness among family members. The baseline severity of depressive symptomatology was measured using the self-report Quick Inventory of Depressive Symptomatology (QIDS) [40].

From January 2008 to December 2012, the ATC assessed 652 patients who were referred to the program by physicians in the community (mostly family physicians). Of these subjects, 238 (72 men and 166 women) presented with a primary diagnosis of MDD, with a current episode that was mild to severe.

### Follow-up assessment

Patients who had been diagnosed with a primary MDD and a current depressive episode (mild to severe) were contacted by a nurse for a follow-up interview between 2 to 5 years after the initial assessment (January 2013 to December 2014). The target follow-up time ranged from a minimum of 24 months to a maximum of 60 months. A minimum of 24 months was chosen because patients who did not remit or recover within this time frame could be considered to have a “chronic” course of depression. The maximum length of follow-up was restricted to 60 months to avoid excessive recall bias. If patients were no longer followed by the ROMHC, they were contacted directly if they had given consent to be contacted for research. Otherwise, their general practitioner was contacted.

With patients’ consent, a follow-up assessment was conducted by a clinical nurse who had substantial experience in mental health, particularly in the mood disorders field, and was trained to administer the Mood Disorder module of the SCID. The psychiatric nurse collected information about changes in demographic data occurring between the intake and the follow-up visit; in addition, information was gathered about relevant clinical events, such as number of hospitalizations owing to mental health issues, suicide attempts, structured psychotherapy, and pharmacological treatment for depression during the follow-up period. The Mood Disorder module of SCID-I was administered to collect information about the presence of mood symptoms during the follow-up interval. Though recall of previous episodes is often a challenge for individuals suffering from depression, every effort was made to ascertain periods of remission of symptoms, recovery, and recurrences after remission or recovery. The chronology of events was registered as accurately as possible using the Life Chart method [41]. For patients who had been treated in the program, written reports of clinicians who treated the patient were used as complementary information on the clinical course. Information about childhood abuse or neglect was collected using the Adverse Childhood Experience (ACE) Questionnaire [42].

### Statistical analysis

Statistical analyses were carried out using the software Statistical Package for the Social Sciences (SPSS), Version 23.0 for Windows (IBM Corp., Armonk, NY, USA).

Univariate statistics (chi-square, t-test) were used to compare demographic and clinical characteristics between patients who participated in the follow-up study and those who did not participate.

For the present analysis, indicators of the course of depression were defined as follows [18]. Recovery was defined as full symptom remission from an MDE for 8 weeks or more; this class included patients with no residual symptoms. Remission included patients who did not meet the full criteria for MDE for 8 weeks or more, but presented with residual symptoms. Recurrence was defined as the development of a new mood episode meeting the full criteria for MDE during recovery or remission. Chronic course was defined as the persistent presence of symptoms meeting the criteria for MDE, without any period of recovery or remission, for 2 years or more after the baseline visit.

Life table survival methods were used to measure time to remission or recovery from an MDE and time to recurrence following remission or recovery from an MDE episode [43]. Estimated cumulative remission and relapse rates were measured using the Kaplan and Meier [44] method. The effect of predictors of time to remission/recovery and first recurrence were tested using bivariate and multivariate Cox proportional hazards regression tests [45], adjusting for age, sex, and length of follow-up. A two-tailed *p* < 0.05 was used for all significance tests.

## Results

### Baseline characteristics and follow-up study

Among the 238 subjects presenting with a primary diagnosis of MDD, and a mild to severe current episode at the initial assessment between January 2008 and December 2012, 119 (83 women and 36 men) participated in the study (50.0%). Participants were slightly older than non-participants: mean (SD) = 44.4 years (12.6) vs. 40.8 years (12.2), respectively. There were no other significant differences in sociodemographic and clinical characteristics at baseline between participants and non-participants (Table 1).

**Table 1:** Sociodemographic and clinical characteristics of participants and non-participants in the follow-up study

Variables at the initial assessment	Participants				
	No *N* = 119	Yes *N* = 119	test	df	p
Age	**40.8 (12.2)**	**44.4 (12.6)**	***t*** **= 2.24**	**236**	**0.03**
Sex (female) (*N*,%)	83 (69.7%)	83 (69.7%)	χ^2^ = 0.00	1	1.00
Education, higher than high school (*N*,%)	49 (45.4%)	61 (51.3%)	χ^2^ = 0.79	1	0.38
Marital Status (N,%)					
Single	40 (33.6%)	43 (36.1%)	χ^2^ = 0.89	2	0.64
Separated/divorced/widowed	20 (24.4%)	33 (27.7%)			
Married/significant other	50 (42.0%)	43 (36.1%)			
Having children (*N*,%)	71 (59.7%)	65 (54.6%)	χ^2^ = 0.62	1	0.43
Working full or part time (*N*,%)	26 (21.8%)	18 (15.1%)	χ^2^ = 1.78	1	0.18
*Clinical variables*					
Initial severity of the MDE (SCID) (*N*,%)					
Mild	20 (16.8%)	24 (20.2%)	χ^2^ = 0.45	2	0.80
Moderate	77 (64.7%)	74 (62.2%)			
Severe	22 (18.5%)	21 (17.6%)			
Age at onset (mean, SD)	23.5 (12.1)	25.2 (11.8)	*t* = 1.07	236	0.29
Duration of MDD^a^ (mean, SD)	17.7 (11.0)	19.3 (12.1)	*t* = 1.04	236	0.30
Length of index MDE^b^ (mean, SD)	40.9 (52.2)	37.7 (48.1)	*t* = 0.50	236	0.62
MDE, chronic	61 (51.3%)	56 (47.1%)	χ^2^ = 0.52	1	0.60
Number of previous MDEs					
None	26 (21.8%)	26 (21.8%)	χ^2^ = 0.77	3	0.86
One to two	26 (21.8%)	29 (24.4%)			
Three to five	25 (21.0%)	20 (16.8%)			
More than five	42 (35.3%)	44 (37.0%)			
Past history of suicide attempts	44 (37.0%)	46 (38.7%)	χ^2^ = 0.07	1	0.79
Family history of mood disorder in first degree relatives	70 (61.4%)	66 (56.9%)	χ^2^ = 0.49	1	0.51
Anxiety disorder, current	72 (60.5%)	67 (56.3%)	χ^2^ = 0.51	1	0.60
Number of comorbidities in Axis III					
None	49 (41.2%)	42 (35.3%)	χ^2^ = 1.73	2	0.42
One or two	39 (32.8%)	37 (31.1%)			
Three or more	31 (26.1%)	40 (33.6%)			
Personality disorder, severe	8 (6.7%)	14 (11.8%)	χ^2^ = 0.17	1	0.19
GAF (mean, SD)	53.3 (5.9)	53.5 (5.2)	*t* = 0.33	236	0.74
*History of childhood maltreatment*					
Emotional abuse	Not assessed	67 (56.3%)			
Physical abuse		47 (39.5%)			
Sexual abuse		42 (35.3%)			
Emotional neglect		75 (63.0%)			
Physical neglect		28 (23.5%)			

^a^Calculated in years; ^b^Calculated in months

*MDE* Major depressive episode, *MDD* Major Depressive Disorder, *GAF* Global Assessment of Functioning, *SCID* Structured Clinical Interview for DSM-IV TR disorders

There were some differences in the percentage of participation among patients with a baseline assessment in different years (2008: 34.1%; 2009: 55.2%; 2010: 63.3%; 2011: 47.9%; 2012: 41.7%), but no linear trend was observed (test for linear trend *p* = 0.77). The most common reasons for not participating were that the patient could not be reached (65%), the patient declined to participate (19%), and the patient agreed to participate but did not attend the interview (8%). Three patients died and the diagnosis was changed for three patients (from MDD to bipolar disorder for two patients and from MDD to schizoaffective disorder for one patient).

Most participants reported pharmacological treatment during the follow-up (99%). The most frequently prescribed psychotropics were antidepressants (95% of the sample), atypical antipsychotics (60%), and benzodiazepines (52%). Cognitive-behavioral therapy was provided to 51% of the sample and interpersonal therapy to 17% of patients. The psychoeducational group program “Wellness and Recovery Action Plan” [46] was delivered to 30% of the patients.

Regarding clinical characteristics (Table 1), about half the sample had an index MDE lasting 2 years or more and about half had a history of three or more previous episodes. Only 6% of patients had a first and non-chronic episode of MDD. Fifty percent of patients had a history of hospitalization for depression. Thirty-eight percent of patients had a history of suicide attempts preceding the index episode.

The median follow-up time was 40.2 months (min.–max. = 23.6–74.4; 25th percentile =29.7, 75th percentile =49.2) for the whole sample (*N* = 119). Fifty-four patients (45.4%) exhibited the full MDE criteria during the whole follow-up period. Twenty-four participants (20.2%) fully recovered, with no additional MDD symptoms for 8 weeks or more, and 41 participants (34.4%) remitted. Among the 65 patients who recovered or remitted, 13 (54.2%) and 12 (29.3%) had one or more subsequent recurrences, respectively. Forty patients (33.6%) recovered or remitted during the 2–5-year follow-up and had no recurrences.

### Survival times

**Fig. 1:**
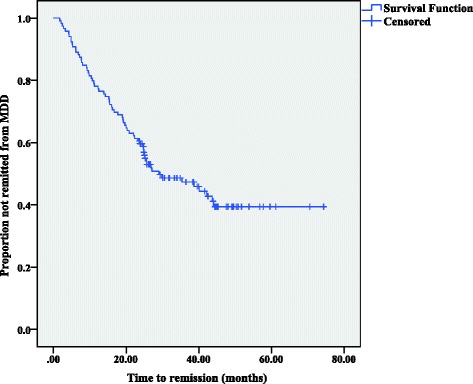
Time to recovery/remission (*N* = 119)

Figure 1 shows the time to remission or recovery for the entire sample. Based on the cumulative probability of recovery/remission calculated by the Kaplan–Meier survival table, the median time to remission/recovery was 28.9 months (95% CI = 16.9–41.0); the probability of achieving recovery/remission within 1 year was 22.7%, within 2 years was 41.3%, and within 3 years was 52.7%.

**Fig. 2:**
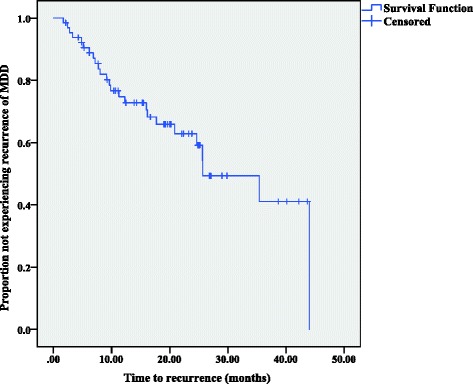
Time to recurrences (*N* = 65)

Figure 2 shows the time to recurrence among patients who achieved remission or recovery (*N* = 65). The median time to the first recurrence was 25.7 months (95% CI = 15.3–36.0); the probability of recurrence within 1 year was 27.2%, within 2 years was 40.8%, and within 3 years was 58.9%.

### Predictors of rate of recovery or remission

Not being married, chronic index MDE, current comorbidity with an anxiety disorder, and childhood history of physical neglect all predicted a slower rate of remission (Table 2). The initial severity of depression was measured using the QIDS during the baseline interviews for 98 patients. Baseline QIDS scores were not significantly associated with the probability of remission or recovery (Table 2).

**Table 2:** Predictors of course of MDD – Cox proportional hazard models, univariate analysis

Predictor at entry^a^	Probability of recovery or remission *N* = 119			Risk of recurrences *N* = 65		
	HR	95% CI	*P* value	HR	95% CI	*P* value
*Sociodemographic*						
Age	0.991	0.972–1.010	0.34	0.975	0.947–1.004	0.09
Gender – female	1.42	0.81–2.49	0.23	2.15	0.74–6.26	0.16
Marital status – single, separated, divorced or widowed	**0.52**	**0.32–0.85**	**0.009**	1.58	0.70–3.54	0.27
Education – Graduate from high school or less	0.82	0.50–1.33	0.42	1.09	0.50–2.41	0.82
Occupation – not working	0.65	0.36–1.31	0.26	0.74	0.28–1.97	0.54
*Clinical*						
Age at onset of MDD	0.992	0.972–1.014	0.49	**0.935**	**0.888–0.985**	**0.01**
Length of MDD	0.997	0.976–1.017	0.75	1.013	0.981–1.045	0.43
Length of index MDE – chronic	**0.45**	**0.27–0.75**	**0.002**	0.41	0.15-1.09	0.07
Severity of index MDE						
Moderate	0.99	0.53–1.85	0.98	1.06	0.39–2.90	0.90
Severe	0.60	0.60–1.40	0.24	0.90	0.21–3.85	0.89
Number of previous MDEs ≥ 3	0.93	0.57–1.51	0.76	**5.28**	**1.95–14.3**	**0.001**
Comorbid anxiety disorder	**0.62**	**0.38–1.01**	**0.06**	0.76	0.34–1.67	0.49
Severe personality disorder	0.68	0.29–1.58	0.37	1.37	0.41–4.63	0.61
History of suicide attempts	0.97	0.59–1.61	0.91	**3.29**	**1.39–7.78**	**0.007**
Number of chronic medical conditions	0.949	0.838–1.074	0.403	1.12	0.93–1.36	0.24
Family history of mood disorder in first degree relatives	1.04	0.63–1.72	0.87	1.61	0.68–3.80	0.28
History of childhood maltreatment:						
Emotional abuse	0.93	0.57–1.52	0.77	1.73	0.75–4.02	0.20
Physical abuse	1.10	0.67–1.81	0.70	1.49	0.67–3.28	0.33
Sexual abuse	0.84	0.50–1.41	0.50	1.02	0.44–2.38	0.96
Emotional neglect	0.88	0.53–1.45	0.62	**3.81**	**1.31–11.10**	**0.01**
Physical neglect	**0.48**	**0.24–0.94**	**0.03**	**2.74**	**1.06–7.05**	**0.04**
Number of Residual symptoms after remission from index MDE	-	-	-	0.989	0.775–1.26	0.93
Baseline QIDS score	0.96	0.90–1.03	0.29	0.99	0.88–1.11	0.81
Length of follow-up	0.99	0.97–1.01	0.40	0.99	0.95–1.03	0.67

Legend: *MDE* major depressive episode, *MDD* major depressive disorder, *QIDS* quick inventory of depressive symptomatology ^a^Reference categories: Gender: male; Marital status: married or common-law; Education: College diploma or University degree; Occupation: working; Length of index MDE: <2 years; Severity of index MDE: mild; Number of previous MDEs: <3; Comorbid anxiety disorder: absent; Severe personality disorder: absent; History of suicide attempts: absent; Family history of mood disorder in first degree relative: absent; Childhood maltreatment: absent

A multivariate Cox regression using those variables associated with the rate of remission at *p* < 0.10 (and adjusting for sex, age, and length of follow-up) showed that the four variables independently predicted rate of remission/recovery (Table 3). The effect size of childhood physical neglect was similar to the effect size of chronic depression (hazard ratio [HR] = 0.43 and HR = 0.45, respectively). The adjustment factors (sex, age, and length of follow-up) were not significantly associated with remission/recovery rate. To determine the possible moderating effects of marital status, length of index MDE, and anxiety comorbidity on the relationship between childhood physical neglect and course of depression, we tested the corresponding interaction terms, which were not significant. The proportional hazards assumption of the Cox model was tested using the −ln(−ln) survival curves ([43], page 165); the assumption was satisfied for all variables entered in the final regression.

**Table 3:** Predictors of course of MDD – Cox proportional hazard models, multivariate analysis

Predictors of time to remission/recovery from index MDE^a^ (*n* = 119)	HR^b^	95% CI	*P* value
Marital status – single, separated, divorced or widowed	**0.53**	**0.32–0.88**	**0.01**
Length of index MDE – chronic	**0.45**	**0.26–0.76**	**0.003**
Comorbid anxiety disorder	**0.60**	**0.36–0.98**	**0.04**
Childhood Physical neglect	**0.43**	**0.22–0.86**	**0.02**
Predictors of time to recurrences^a^ (*N* = 65)			
Age at onset of MDD	0.97	0.911–1.03	0.29
Length of index MDE – chronic	0.48	0.17–1.33	0.16
Number of previous MDEs ≥ 3	**3.91**	**1.26–12.1**	**0.02**
Childhood Emotional neglect	**3.69**	**1.13–11.9**	**0.03**
Childhood Physical neglect	1.15	0.38–3.41	0.81

Legend: *MDE* major depressive episode, *MDD* major depressive disorder ^a^Reference categories: Marital status: married or common-law; Length of index MDE: <2 years; Comorbid anxiety disorder: absent; Length of index MDE: <2 years; Number of previous MDEs: <3; Childhood neglect: absent ^b^Adjusted for sex, age and length of follow-up

### Predictors of recurrence (*N* = 65)

Age at onset of MDD predicted the risk of recurrence; a greater age at onset of MDD was associated with a slower rate of recurrence. Conversely, having three or more previous MDE episodes and a history of suicide attempts preceding the index MDE significantly increased the rate of recurrence. The history of both emotional and physical neglect was associated with faster rates of recurrence. The initial severity of depression was measured using the QIDS during the baseline interview for 56 patients. Baseline QIDS scores did not significantly predict the probability of remission or recovery (Table 2).

The variables associated with the rate of recurrence at *p* < 0.10 were entered into a multivariate Cox proportional regression analysis, adjusting for sex, age, and length of follow-up (Table 3). History of suicide attempts could not be entered into the analysis simultaneously with history of three or more previous MDEs; these two variables were strongly correlated, (concordance =70.8%, kappa =0.42), causing a collinearity problem. The model that included the presence of previous MDEs showed a better fit than the model that included history of suicide attempts (−2 log likelihood =150.56 and 152.54, respectively), and is shown in Table 3. One or more previous MDEs and a history of childhood emotional neglect independently predicted the time to recurrence. The adjustment factors (sex, age, and length of follow-up) were not significantly associated with the risk of recurrence. To determine the possible moderating effects of the presence of previous MDEs on the relationship between childhood emotional neglect and course of depression, we tested the corresponding interaction term, which was not significant. The proportional hazards assumption of the Cox model was tested using the −ln(−ln) survival curves ([43], page 165); the assumption was satisfied for all variables entered into the final regression.

The alternative model showed that the effect of suicide attempts on the risk of recurrences was independent of age, sex, length of follow-up, age at onset of MDD, history of chronic index MDE, and childhood neglect (previous suicide attempts: HR = 2.86, 95% CI = 1.08–7.56, *p* = 0.04; childhood emotional neglect: HR = 4.35, 95% CI = 1.32–14.32, *p* = 0.02).

### Effects of treatment delivered during the follow-up on the rate of recovery/remission

We examined the effects of pharmacological treatment and psychotherapy on the outcome to test if the effect of the predictors on the outcome was influenced by the delivery of a specific treatment. A Cox regression analysis with specific pharmacological treatment (atypical antipsychotics) or specific psychotherapy (Cognitive Behavioral Therapy, Interpersonal Therapy, Wellness Recovery Action Plan) as independent variables did not substantially change the results.

## Discussion

Our results are quite consistent with some previous findings on the course of depression in treatment-resistant patients, or in samples drawn from psychiatric settings, with a minimum follow-up length of 2 years [1, 2]. Similar to studies of tertiary care populations, we observed a large number of patients who did not recover or remit from the index depressive episode during the follow-up (45.4%). Among patients who remitted, 38.5% had one or more recurrences. About 34% of our sample recovered or remitted without any recurrences during the 2–5-year follow-up.

As expected, the course of depression in our population was poorer than in the general population or primary care population, in which the prevalence of a chronic course is about 15%–17% [3]. The present results probably reflect the characteristics of our population, who mostly showed a treatment-resistant course of depression, with a past history of chronic or highly recurrent depression. Kiloh et al*.* [6] found that only 20% of 193 patients admitted to a psychiatric unit with depressive symptoms and followed-up for 15 years recovered and were continuously well; in the same study, 19% of patients remained incapacitated by the illness or committed suicide. The corresponding figures at 25 years were 12% (recovered and continuously well) and 4% (remained incapacitated during the follow-up or committed suicide), respectively [14]. It is often observed that the percentage of patients who do not recover during the follow-up period and the percentage of patients who experience stable recovery tend to decrease as the length of follow-up increases, as the probability of first recovery and the risk of recurrences both increase with time. Kennedy & Paykel [15] found that 71% of patients who recovered from severe depression had recurrences in the 8–10-year follow-up. Mueller et al*.* [13] found that 62% of those ill for the first 5 years had not recovered within the next 5 years. These results suggest that more research should focus on treatment-resistant depression to find more effective treatment for this challenging population. In recent years there have been some encouraging results, which have indicated that specific treatments may lead to improved outcomes. In the pharmacological field, ketamine has shown a superior efficacy for treatment-resistant depression compared with other pharmacological interventions [47]. In the treatment of chronic depression, the cognitive-behavioral analysis system of psychotherapy has produced moderate-to-high effect sizes when compared with treatment as usual and interpersonal psychotherapy (see the recent meta-analysis by Negt et al*.* [48]) and has similar effects to antidepressant medication [49].

Among sociodemographic factors, marital status predicted the course of depression in our study. This result agrees with the few studies that have found that marital status plays a role in predicting the course of depression [4, 13, 50]. Previous studies have shown that MDE length prior to study entry is a consistent predictor of chronic depression [51–53], as is the presence of a greater number of previous MDEs, which also predicts recurrence after recovery (for literature reviews see [8, 9]). We found that earlier age at MDD onset predicted higher risk of recurrences, which is consistent with previous studies [54, 55]; however, its effect could be explained by other variables, as it was not significant in the multivariate model. The finding that comorbid anxiety negatively affected the course of depression, predicting a slower rate of remission, is consistent with previous findings that comorbidity on Axis I is associated with poorer prognosis [8, 9]. A history of suicide attempts predicted a higher risk of recurrences in our sample; however, related previous findings are rather heterogeneous. Two studies found a positive and significant association between suicidality and risk of recurrences [56, 57]. Conversely, another study found that the risk of recurrences was enhanced for those who had not engaged in parasuicidal behavior during the index episode [58] and other studies have failed to find an association [59–61]. In our sample, the strong association between history of suicide attempts and previous MDEs prevented us from considering the independent effect of the two variables.

We found that the delivery of specific treatment was not associated with the course of depression. However, because of the naturalistic study design and the absence of randomization, we cannot draw any conclusions about treatment efficacy in our population. Furthermore, the chronology between the administration of pharmacological treatment and the occurrence of remission or recurrences was not available. However, we can conclude that the effect of the predictors on the outcome was independent of the treatment received during the follow-up.

We found that physical neglect as measured by the ACE questionnaire predicted a slower rate of remission/recovery and emotional neglect predicted a recurrent course of depression. The ACE questionnaire explores childhood adverse experiences in the first 18 years of life. Based on ACE responses, the presence of childhood physical neglect is assumed if the participant estimates that his/her caregiver failed to provide adequate nutrition, clean clothes, protection, or failed to take care of him/her owing to substance use. The presence of emotional neglect is assumed if the participant reports not feeling loved or perceives lack of family love and support [42]. As expected, we found that most patients with a history of physical neglect also reported a history of emotional neglect. Our finding that childhood neglect predicts the course of depression is consistent with previous studies. Brown et al*.* [25] found that maternal lack of affection predicted adult chronic depression in daughters. Wiersma et al*.* [30] found that longstanding experience of emotional neglect was associated with chronic depression. Other studies have assessed neglect as part of a global trauma index, so have not reported its specific role [27, 31, 32]. Therefore, this is the first study to report a specific relationship between physical neglect and slower rate of remission, and to show that emotional neglect predicts a higher rate of depression recurrence. Most of our patients with physical neglect had also experienced emotional neglect, suggesting that patients with physical neglect have a more severe form of neglect. These patients reported that they not only lacked childhood love and support, but also lacked the material nurturance to survive. We can hypothesize that depressed patients with a history of both childhood physical and emotional neglect lack the ability to recover from feelings of deprivation and abandonment, which are often associated with a history of childhood maltreatment, because of the difficulty of experiencing positive and nurturing relationships in adulthood; this perpetuates the absence of a positive view of self and others. Conversely, depressed patients who have experienced only childhood emotional neglect could remit faster, but they remain vulnerable to repeated depressive recurrences. As previous studies have not differentiated between types of neglect [25, 30], we could not directly compare our results with previous findings. Future research is therefore needed to validate the distinct effects of childhood physical and emotional neglect.

Our data highlight the importance of considering childhood neglect as a predictor of course of depression. Data from the United States, Canada, and the UK indicate that more children suffer from neglect than from physical and sexual abuse combined [62–64]. Despite this, a history of neglect is less often recognized than a history of abuse, possibly because a lack of care is more difficult to identify than a history of adverse events; furthermore, neglect has been studied less often than other kinds of maltreatment [65]. We wonder whether the lower recognition of childhood neglect may increase its impact on the course of depression in adulthood, particularly in severely depressed or treatment-resistant patients.

Contrary to other studies [25, 29, 30, 33, 37], we did not find any significant association between childhood abuse and the course of depression during follow-up. There are some possible explanations for this discrepancy, such as differences in methodology and in the populations studied. We used the ACE, which features yes/no questions and therefore (unlike some other questionnaires) cannot detect different degrees of severity or frequency of specific kinds of abuse. Consequently, we could not differentiate more severe or more prolonged abuse from less severe or less frequent abuse, possibly diluting its effect. Wiersma et al*.* [30] found that the presence of abuse that occurred “regularly/often/very often,” but not abuse that occurred “once/sometimes” differentiated between chronic and non-chronic depression. It may be less important to differentiate neglect, which is defined as an ongoing long-term situation. A second explanation is that neglect, but not abuse, predicts the course of depression in treatment-resistant depressed populations. As neglect is recognized less often, it may be less often treated with specific therapy. However, we cannot confirm this hypothesis because of the paucity of studies of tertiary care samples.

Our results suggest that the history of childhood neglect predicts the course of depression, independent of the past history of depression. Hovens et al*.* [66] found that the relationship between childhood adversity and course of depression or anxiety in adulthood was mediated by the severity of depression at a young age (20s). In our study, the relationship was independent of the effect of depressive severity at intake, as measured by the SCID or by the self-rated QIDS. The relationship was also independent of other predictors of course of depression, such as marital status, length of the index MDE, comorbidity with an anxiety disorder, age at onset of MDD, or number of previous episodes.

Several possible mediators of the relationship between childhood trauma and symptoms of depression have been suggested, such as maladaptive schemas of vulnerability to harm and self-sacrifice [67], negative cognitive styles with fear of criticism and rejection [68], hopelessness [69], affect dysregulation (see for a meta-analysis [70, 71]), and rumination [72]. These factors, which were not considered in our study, may mediate the relationship between childhood maltreatment and course of depression. Recent studies have shown that exposure to childhood maltreatment predicts poorer response of MDD to pharmacological treatment [73], predicts a better response to psychotherapy than to pharmacotherapy [74], and predicts the response to specific psychotherapies [75]. These results emphasize the importance of the clinical assessment of childhood maltreatment in selecting patients who may require specific interventions focused on maltreatment history. More research is needed to elucidate the neurophysiological and epigenetic mechanisms that connect childhood trauma to course of depression to identify more effective pharmacological treatments [76, 77].

### Limitations of the study

Although we did not find any significant differences in participants and non-participants, only 50% of the initial sample participated in the follow-up.

The study was limited because it was a retrospective follow-up. Evidence suggests that childhood adverse events may be underreported by adults; furthermore, the relatively low reliability of reports of childhood maltreatment by adults may lead to doubts about their validity [78]. The ACE instrument has satisfactory test–retest reliability [79]; however, there are no data on its validity in retrospective studies. Errors in the retrospective reports of childhood experiences may be a result of low reliability and validity of autobiographical memory in general or the presence of specific biases related to mood state [80]. This last source of error could cause a systematic bias in the analysis of the effect of childhood adverse experiences on the severity and course of depression. A tendency in severely depressed patients to exaggerate or misrepresent their childhood (to describe it as more traumatic than it was) would overestimate the risk (and vice versa if individuals tend to suppress negative memories). However, some evidence suggests that recall bias associated with the mood state at the time of the interview is unlikely to explain our results. Although recall bias may invalidate retrospective study findings, two literature reviews [78, 81] have concluded that recall of childhood experiences is unlikely to be distorted by depressed mood. Fergusson et al*.* [82] examined the reliability of reports of childhood sexual and physical abuse at 18 years and 21 years and found that, although the consistency between the two reports was relatively low (kappa of about 0.45), the errors were not correlated with measures of psychiatric status, and the relative risk of having a mood disorder associated with childhood abuse was reasonably robust to reporting errors. In two studies that examined the reliability of the Parental Bonding Instrument, measures of parental representations were quite stable for long periods of time in depressed patients, despite changes in the level of depressed mood [83, 84]. Brown et al*.* [85] analyzed the validity of retrospective recall of childhood experiences using the Childhood Experience of Care and Abuse instrument; these authors found that depressed patients have a tendency to underreport, rather than overreport, childhood maltreatment. Two longitudinal prospective studies have validated the association between childhood maltreatment and poor course of depression [26, 27, 86].

There is also some evidence that depressed patients tend to underreport past depressive episodes, with greater recall failure over time [87]. The Life Chart, used to retrospectively assess the course of depression, has proved to be a reliable instrument that can reduce recall bias [88].

A final limitation was that we did not use a structured family history interview to record participant family history, which may have reduced the reliability of our data [89]. However, highly experienced staff collected collateral information about mood disorders affecting first-degree relatives.

## Conclusions

We found that childhood neglect predicted the course of depression independently of demographic and clinical factors. This suggests the importance of investigating the presence of childhood maltreatment in the clinical assessment of patients with MDD to identify individuals at risk of poorer prognosis. Further research is needed to elucidate which mediators explain the association between childhood maltreatment and course of depression, and to investigate whether specific treatments may improve the prognosis and the quality of life of individuals with a history of childhood maltreatment and current depression.

## Abbreviations

ACE: Adverse Childhood Experience
ATC: Assessment and Treatment Clinic
DSM: Diagnostic and Statistical Manual of Mental Disorders
HR: Hazard Ratio
MDD: Major Depressive Disorder
MDE: Major Depressive Episode
QIDS: Quick Inventory of Depressive Symptomatology
ROMHC: Royal Ottawa Mental Health Centre
SCID: Structured Clinical Interview for DSM-IV-TR
